# Parents’ perceptions of parental consent procedures for social science research in the school context

**DOI:** 10.1080/13645579.2023.2222539

**Published:** 2023-06-21

**Authors:** Thabo J van Woudenberg, Esther Rozendaal, Moniek Buijzen

**Affiliations:** a Erasmus School of Social and Behavioural Sciences, Erasmus University, Rotterdam, the Netherlands; b Behavioural Science Institute, Radboud University Nijmegen, Nijmegen, the Netherlands

**Keywords:** Parental consent, active consent, passive consent, school-based research, vignettes

## Abstract

Typically, parents or other legal guardians are asked for an active declaration that the participation of their child in scientific research is informed and voluntary. However, asking for active parental consent leads to lower quality studies and passive parental consent might be preferable. In this study, we used an online survey in which parents (*N* = 156) watched video vignettes of multiple types of research in the classroom and asked them to rate the appropriateness of using active and passive parental consent. The results indicated that parents perceived active consent procedures as more appropriate in most types of research. However, particularly for secondary school children passive consent was rated as comparably appropriate for several types of research (e.g. observation and questionnaire studies). Other aspects of providing consent are displayed in a supplementary online dashboard. We conclude with recommendations for parental consent procedures for social science research in the school context.

The guiding principle for the ethical conduct of research with human subjects is informed consent (Rothstein & Shoben, 2013). In the vast majority of social science studies, researchers are obliged to obtain informed consent from the participants in which they declare that their participation is informed and voluntary (Burgess, 2007). When the participants in a study are minors, they are not considered capable of confirming that they fully understand what the study is about and acknowledge that their participation is voluntary. In these cases, someone with parental responsibility such as parents, guardians or designated authority (from here on referred to as *parents*) must provide consent before participation in the study. We call this *parental consent* (Manti & Licari, 2018). However, legislation about collecting personally identifiable information from minors, which applies to most social science research, varies across the globe. For example, the United States COPPA (Children’s Online Privacy Protection Act, 2013) mandates that collecting personal information from children under the age 13 requires parental consent. In Europe, the GDPR (General Data Protection Regulation, 2016) prescribes that parental consent is mandatory for children younger than 16 years old. However, member states that are covered by the GDPR are allowed to maintain a minimum age of 13 years, which happens for example in Belgium, Poland, Sweden, and the United Kingdom.

Generally, Institutional Review Boards (IRBs) require that researchers use *active* parental consent procedures (Ellickson & Hawes, 1989; Esbensen et al., 1996). In this procedure, parents of prospective participants actively indicate their willingness for their child’s participation in the study, for example by returning a signed consent letter or filling out an online form. Under active consent procedures, children for whom no consent is returned, or whose parents explicitly decline to provide consent, are not included in the study (Ellickson & Hawes, 1989). Subsequently, the researcher must monitor the participants for any verbal or non-verbal clues that they may disagree or wish to stop participating in the study (Field & Behrman, 2004). However, asking for active parental consent come with several disadvantages.

First, active parental consent procedures lead to smaller samples. In a meta-analysis of 13 empirical studies in school settings, the response rate for passive consent procedures was significantly higher than that for active consent procedures (Liu et al., 2017). In one study (Horn et al., 2009), the passive consent sample was even five times larger than the active consent sample. Although drop-out rates are higher in passive consent samples, the total number of completed participation in passive consent procedures exceeds that of active consent procedures (Spence et al., 2015). The consequence of smaller sample sizes is smaller power and reduced reliability and quality of the conclusions of the research (Button et al., 2013). Unfortunately, the power of published studies in social sciences is still at an alarmingly low level (Szucs et al., 2017).

Second, active parental consent procedures lead to systematically biased samples. The same meta-analysis (Liu et al., 2017) demonstrated that using an active consent procedure leads to an underrepresentation of minorities (Dent et al., 1993; Esbensen et al., 1999; Horn et al., 2009; Unger et al., 2004), as well as male (Courser et al., 2009; Jelsma et al., 2012; Pokorny et al., 2001; Unger et al., 2004) and older participants (Courser et al., 2009; Pokorny et al., 2001). In addition, psychosocial and behavioral outcomes differ between the samples of active and passive consent procedures (Liu et al., 2017). As well as being ethically undesirable, this is problematic because biased samples lead to an inaccurate representation of the studied topic and biased estimates of the studied effects (Dent et al., 1993; Esbensen et al., 1996). The consequence of low external validity is that behavioral regulations and interventions may only be applicable to the overrepresented group, not work to their full potential, or not be effective in reality at all (Shaw et al., 2015). So again, active consent can lead to a decrease in the quality of the research.

Third, active parental consent procedures require higher investment (in terms of energy, time, and money) from parents, children, schools, and researchers, raising both principal and practical issues. When a parent does not respond in an active consent procedure, the researcher has to interpret this as the refusal for the child’s participation in the study. The child therefore cannot participate in the study. However reasons for not responding might be found in the required time and energy investment. A previous study investigating non-responding parents in an active consent procedure in a survey showed that failure to return the consent form was around ten times more likely to reflect latent consent than latent refusal (Ellickson & Hawes, 1989). Not only does this suggests that equating non-response in active consent procedures with non-consent is an incorrect conclusion most of the time, but it also means that in total more parents will have to return a form to communicate their consent. Moreover, it means that more children must return the consent forms, involving a larger time investment for the children, schools, and researchers. Similarly, attempting to raise the response rate in active consent procedures requires more time and money investment from researchers. Ellickson and Hawes (1989) also showed that active consent procedures take up more of the researchers’ working hours and are more expensive, estimated at $15.00 per participant at the time of that paper. Corrected for inflation, this would imply an additional cost of $28.32 per participant in 2022. This is problematic because the workload in schools and academia is high and financial resources for research projects are often scarce.

Adding up these disadvantages associated with active parental consent procedures has the consequence that some researchers believe that all future studies should employ a *passive* consent procedure in school-based research (Dent et al., 1993; Ellickson & Hawes, 1989; Manti & Licari, 2018). In this procedure, parents of prospective participants are informed about the study and are given the opportunity to indicate their willingness for their child to *not* participate in the study. Under passive consent procedures, only children for whom the parents explicitly decline to provide consent are not included in the study. But, the risks of passive parental consent are that the researcher never is certain that the parent received the notification, understood the information, and was able to exclude the child successfully when preferred (Esbensen et al., 1996). In addition, academics have not yet reached a consensus about this dilemma, given that institutional review boards vary in their decision to accept passive consent procedures as adequate (Higgerson et al., 2014). Given these substantial considerations and varying precedence, deciding between active or passive parental consent procedures can be a delicate consideration for researchers and institutional review boards. We believe that parents are important stakeholders in this dilemma and can help us to make better decisions in this regard. Therefore, we investigated their perspective regarding whether active consent procedures should be followed or whether passive consent procedures suffice for social science research in the school context.

## The current study

The goal of this study was to include the parents’ perspective on the most appropriate consent procedure for research in the school context. To study the parents’ perspective, we used an online survey in which parents were exposed to multiple types of research in video vignettes and were asked to indicate how they rated active and passive parental consent in terms of appropriateness for the type of research. Based on the benefits of passive consent outlined previously, we were interested in whether passive consent is rated as appropriate as, or more appropriate than, active consent. To test this, we used a reversed inferior hypothesis (Lakens et al., 2018). Specifically, we tested whether active parental consent is rated as more appropriate than passive parental consent (BF_10_), but considered the strength of the evidence for this not being the case. In short, we were looking for support for the null hypothesis that active consent is not perceived as more appropriate than passive consent (BF_01_).

## Method

### Design

We used an online panel test our hypotheses in a diverse sample. Specifically, we used video vignettes to investigate the perceived appropriateness of both forms of consent for a variety of research methods in the school context (i.e. observational, focus group, co-creation, interview, diary, questionnaire, longitudinal questionnaire, data donation, objective measures, and clinical studies). The list of research methods was achieved by combining the most common qualitative and quantitative research methods in the school context as well as contemporary research methods of interest to our field. Moreover, we added the clinical studies vignette because active parental consent is mandatory and it served as a yardstick for gauging parental preferences against current consent legislation. Each vignette briefly described the research method in terms of the role of the researcher and the type of data gathered. The vignettes were subtitled in Dutch and English to accommodate non-native speakers and hearing impaired, and were supported with illustrations created for this study. The procedure was approved by the local ethical committee of the Erasmus University (Ethics application number: ETH2122–0274). The scripts and links to the video vignettes can be found on the Open Science Framework (https://osf.io/ekagz/).

### Participants and procedure

Before collecting the data, we preregistered our target sample size. Because we had no specific expectations about the effect sizes in this study, we were not convinced we could perform an accurate a priori sample size analysis. Instead, we used resource constraints to justify our sample size (Lakens, 2022). In coordination with the panel agency, we estimated that we could collect data from a maximum of 250 participants, given our budget and the number of panel members with children in the target group. However, we could potentially draw our conclusions with a smaller sample. Therefore, we registered two checkpoints (at *N* = 150 and *N* = 200) and checked whether we had found at least moderate evidence for all the vignettes. At the second checkpoint, we had gathered enough evidence to discontinue data collection at an early stage.

Data were collected between April 7th and 12th, 2022. The recruitment and compensation of the participants were managed by the panel agency PanelClix. The agency purposefully approached members of the online panel who had at least one child in the target group of children in the last three years of primary school or the first three years of secondary school. This corresponds approximately to children between the ages of 8 and 16 years old. These members were invited to participate in an online survey with an estimated completion time of 20 minutes and would be compensated with points that they could save and later redeem for money, depending on the total number of points they had saved.

Interested members were redirected to an online survey in Qualtrics. The initial 211 participants received detailed information about the study and data collection and consented to voluntary participation in the study. Subsequently, participants received a screening question asking whether they had at least one child within the target group. Three participants replied that they did not and were excluded from participation. After an inspection of the ages and corresponding classes of the children, an additional four participants were excluded from the sample. Participants with multiple children within the age range were asked to select one of their children and keep this child in mind when answering the questions.

Participants watched the introduction video and were instructed how to respond to the 10 video vignettes. After watching the vignettes, the participants completed the additional questions about specific topics as well as passive consent, cluster consent, communication with parents, rewards for participants, and the sharing of the data. The survey ended with queries about the demographical information of the participants.

In addition, we preregistered to screen for inattentive respondents by looking at speeding and failing the instructed response question (Kees et al., 2017). Before distributing the survey, we registered the benchmark completion time of eight minutes, but none of the participants met this exclusion criterion. Also, we included an instructed response item. Near the end of the survey, participants were presented with a directed instruction to select the *totally disagree* answer (Gummer et al., 2021). 48 participants failed to select this answer and were excluded from the analyses.

In the analytical sample (*N* = 156, *M*_*age*_ = 44.80, *SD*_*age*_ = 6.58), 73 (46%) participants identified themselves as male, and 149 (95.51%) were born in the Netherlands. The ages of the reported children ranged from 8 to 16 years old (*M*_*age*_ = 11.80, *SD*_*age*_ = 1.95), 85 (54.48%) attended primary school and 80 (51.28%) were boys.

### Measures

#### Perceived appropriateness of active and passive parental consent

The participants indicated how appropriate both active and passive parental informed consent was for each vignette. Participants could rate the appropriateness on a 7-point slider scale, ranging from *not appropriate at all* (1) to *very appropriate* (7). The ratings for active and passive consent were requested on two separate pages, and each time the illustration of the vignette was presented alongside the research method. To help guide the participants, the scale was indicated by seven faces, ranging from a very sad face to a very happy face (based on Holder et al., 2010). The participants responded by selecting the face that best matched their response. Even though it is arguable that this results in a ordinal scale (Kuzon et al., 1996), we regard this variable as an ‘imperfect interval scale’ (Borgatta & Bohrnstedt, 1980). That is, we acknowledge that this is a crude measurement of the latent construct, but assume that this measure approaches an interval scale. On average, the respondents rated active parental consent a score of 5.31 (*SD* = 1.66) and passive parental consent a score of 4.10 (*SD* = 2.04).

#### Exploratory variables

Furthermore, we asked an additional set of questions of particular interest to our research group. Because these results can be helpful for other researchers, these are also briefly reported in this study. The first set of statements investigated how appropriate passive parental consent is for questionnaire studies in which questions are asked about: the domestic situation, mental well-being, friendship nominations, and personally identifiable information. In addition, we added a separate statement on how appropriate passive parental consent is when children are asked to create media content (photos or videos) in a co-creation or diary study. Participants responded to these statements on a 7-point Likert scale ranging from *totally disagree* (1) to *totally agree* (7). Again, the scale was treated as an ‘imperfect interval scale’.

Next, the participants were shown a video about a specific format for obtaining parental consent for multiple studies within one school year. In this cluster consent format, parents are asked to provide active parental consent at the start of the school year for a series of studies on a determined topic. For each study within the school year, parents are informed and have the opportunity to opt out. Participants in the current study were asked to respond on this format in general and whether this was appropriate to use for the 10 research methods.

Moreover, participants were asked whether they have been approached previously to provide consent for their child to take part in academic research. Follow-up questions asked why people did not respond previously. In addition, participants were asked via which channel(s) they would like to consent (or opt out) to a study, and what they thought a reasonable reward would be for participation.

The last items investigated the opinions about sharing the research data. Specifically, participants were shown several statements about sharing the data with the other participants, other researchers, with the article and sharing the data in a repository for others to use. For each statement, participants indicated how problematic this was for them on a 7-point Likert scale, ranging from *totally disagree* (1) to *totally agree* (7). The scores were reversed so that a higher score meant more open to sharing the data and treated as ‘imperfect interval scale’.

### Transparency and openness

Before data collection, we registered the hypothesis and analysis plan on the Open Science Framework (https://osf.io/3qp4n), in addition to the vignettes, dependent variables, exclusion criteria, and all secondary measures in the study, as well as how we determined the sample size. All data and supportive materials are archived on the Open Science Framework (https://osf.io/ekagz).

### Plan of analysis

The data involved in this study were cleaned and analyzed in R, version 4.0.2 (R Development Core Team, 2013). To test the main hypothesis regarding whether passive parental consent was not perceived as less appropriate than active parental consent, we used a reversed inferior test (Lakens et al., 2018). We defined a one-sided t-test with the alternative hypothesis that active parental consent was viewed as more appropriate than passive parental consent. However, to confirm our hypothesis we were interested in the amount of evidence for the null hypothesis (BF_01_), meaning that we would accept our hypothesis when the Bayes factor (BF) for the alternative hypothesis was smaller than 1/3 (BF_10_ < .33; BF_01_ >3).

To test the differences between the appropriateness of active and passive parental consent a Bayesian t-test was used from the BayesFactor package (Morey & Rouder, 2022). Default objective priors were used (i.e. Cauchy prior with a width of 0.707). The sensitivity of the prior width was assessed with the Bayes factor robustness checks in JASP (JASP Team, 2017). Each research method was analyzed separately.

## Results

### Testing the hypothesis

**Figure 1.**
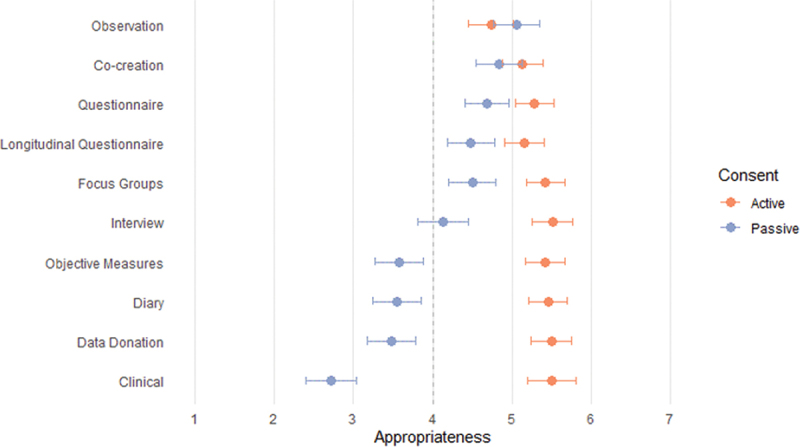
Mean scores of appropriateness of active and passive parental consent for ten research methods.

Note: Error bars indicate 95% confidence intervals.

Figure 1 displays the mean ratings and confidence intervals for active and passive parental consent per each research method. What stands out from this figure is that, on average, active parental consent was rated as very appropriate, with most scores between 5 and 6. In addition, for half of the research methods (*observation*, *focus group*, *co-creation*, *questionnaires*, and *longitudinal questionnaires*), passive parental consent scored on average above the midpoint of the scale. Moreover, the confidence intervals for *observation* and *co-creation* overlapped, indicating similar preferences for passive and active consent. Similarly, the differences between active and passive consent were relatively small for *focus groups*, *questionnaires*, and *longitudinal questionnaires*. For the bottom five research methods, the differences were bigger. The mean rating for passive consent for *interviews* was slightly above the midpoint of the scale, but the confidence interval includes the midpoint. For the other four (*diary*, *objective measures*, *data donation*, and *clinical studies*), the mean ratings for passive consent fell noticeably below the midpoint of the scale. Passive consent for *clinical* studies was perceived as very inappropriate, which is in line with expectations, considering that active parental consent is mandatory by federal law for this type of research in the country where this study was conducted.

Table 1 shows the mean ratings for both active and passive parental consent for the ten research methods. For most research methods we found support for the alternative hypotheses, indicating that parents consider active parental consent more appropriate than passive parental consent. However, a different pattern emerged for observation and so-creation. Observation showed strong evidence for the null hypothesis (BF_01_ = 25.71 ± 0.00%). Given these data, it is 25 times more likely that active consent is not considered more appropriate than passive consent for observational studies, compared to the alternative hypothesis that active consent is more appropriate. A follow-up t-test showed moderate evidence that active and passive consent were statistically equivalent (BF_01_ = 4.32 ± 0.00%). Similarly, co-creation yielded anecdotal evidence for the null hypothesis (BF_01_ = 2.48 ± 0.00%), and the follow-up t-test showed moderate evidence that active and passive consent were statistically equivalent (BF_01_ = 4.52 ± 0.00%).

**Table 1.** Mean scores, Bayes factors, conclusions of difference, and standardized effect sizes for the appropriateness of active and passive parental consent for ten research methods.

Research Methods	Passive [95% CI]	Active [95% CI]	BF_10_	Conclusion	Cohen’s D
Observation	5.06 [4.77, 5.36]	4.74 [4.45, 5.02]	0.04	Pas = Act	.11
Co-Creation	4.84 [4.55, 5.13]	5.13 [4.88, 5.39]	0.40	Pas = Act	.11
Questionnaire	4.69 [4.41, 4.96]	5.29 [5.05, 5.53]	10.44	Pas < Act	.23
Longitudinal Questionnaire	4.48 [4.18, 4.78]	5.16 [4.91, 5.41]	27.40	Pas < Act	.26
Focus Group	4.50 [4.2. 4.80]	5.42 [5.18, 5.66]	>100	Pas < Act	.34
Interview	4.13 [3.82, 4.45]	5.51 [5.26, 5.76]	>100	Pas < Act	.49
Objective Measures	3.58 [3.27, 3.89]	5.42 [5.17, 5.68]	>100	Pas < Act	.66
Diary	3.54 [3.24, 3.85]	5.46 [5.21, 5.70]	>100	Pas < Act	.71
Data Donation	3.48 [3.17, 3.79]	5.50 [5.25, 5.75]	>100	Pas < Act	.73
Clinical	2.72 [2.41, 3.04]	5.51 [5.2. 5.81]	>100	Pas < Act	.92

Therefore, we conclude that parents perceived (1) active and passive consent as equally appropriate for *observational* and *co-creation* studies, (2) passive consent as slightly less appropriate but still acceptable as there is a 95% chance that the mean is above the midpoint of the scale for *questionnaire, focus groups*, and *longitudinal questionnaire*studies, and (3) active consent as favored for *interview, diary*, *objective measures*, *data donation*, and *clinical studies.*

### Exploring differences

We conducted several exploratory analyses to investigate potential differences in the ratings for active and passive consent. First, we looked at whether parents who had been invited in the past to provide consent for their child(ren) rated passive and active consent differently to those who had not previously been invited. A Bayesian mixed-effects model indicated moderate evidence (BF_10_ = .15 ± .98%) that there was no difference in the acceptability scores. We also considered the sex and the age of the parent. Again, the Bayesian mixed-effects models indicated moderate to strong evidence that there were no differences in acceptability scores based on the sex (BF_10_ = 0.20 ± 1.7%) or age (BF_10_ = 0.08 ± 0.84%) of the parents.

Next, we looked at the sex and the school level of the child. A Bayesian mixed-effects model indicated moderate evidence (BF_10_ = .10 ± 4.4%) that there was no difference in acceptability scores for boys or girls. Moreover, we found strong evidence that there was no difference in scores in general for primary and secondary school children (BF_10_ = .08 ± 1.02%). However, we identified anecdotal evidence (BF_10_ = 2.88 ± 0.93%) that the differences between active and passive consent were bigger in primary school (*M*_*active*_ = 5.54, *M*_*passive*_ = 3.91, *M*_*difference*_ = 1.54) than in secondary school (*M*_*active*_ = 5.15, *M*_*passive*_ = 4.34, *M*_*difference*_ *=* 0.81). Splitting the vignettes into primary and secondary schools also provided new insights. Specifically, we ran the same Bayesian paired samples t-tests from the preregistered analyses for the primary and secondary schools separately. For the primary school children, no different conclusions were drawn. For children in secondary school (*n* = 71), we found anecdotal to moderate evidence that parents did not think active consent to be more appropriate for *focus groups* (BF_01_ = 2.63 ± 0.00%), *questionnaire* (BF_01_ = 2.08 ± 0.00%), or *longitudinal questionnaire* (BF_01_ = 3.01 ± 0.00%) studies. Based on these results, we can tentatively state that parents of secondary school children do not think that active parental consent is more appropriate than passive parental consent for *observational*, *co-creation*, *questionnaire*, *longitudinal questionnaire*, and *focus group* studies.

### Descriptive results on consent procedures

Given our numerous descriptive results, we created a dashboard to present these results effectively (see https://thabovw.shinyapps.io/consent_dash/). In this article, we are only able to pay extra attention to some of the descriptive results. Here we want to highlight that passive parental consent was less acceptable when the questions in the questionnaire pertained to personally identifiable information (*M*_passive_ = 3.60), or when their children would take pictures or create videos (*M*_passive_ = 3.57). Also, the idea of clustering the consent (i.e. providing active consent for research in general and passive consent per specific study) was rated as highly appropriate in general (*M* = 5.45), but parents had their reservations in relation to *automatic measures*, *focus groups*, *data donations*, and *clinical studies*.

Moreover, participants were asked via which channel(s) they would like to consent to (or opt out of) a study, and what they thought represented a reasonable reward for participation. Via *email* (64.10%; 100/156) and a *website* (54.49%; 85/156) were the most selected channels. In a follow-up question, most of the participants agreed that it would be important to use the schools’ existing channels of communication. In addition, the majority of the parents indicated that a *gift for the class* (42.31%; 66/156) or a *gift for the child* (34.62%; 54/156) were the most fitting rewards for participation. A substantial number of parents believed that no reward was necessary (31.41%; 49/156). When parents suggested any kind of monetary reward, they considered €5.00 per hour as reasonable. This is considerably higher than the minimum wage of 15 year old’s (€ 3,04 per hour) in the Netherlands.

The last items pertained to open science practices, investigating parents’ opinions about sharing anonymized research data with the other participants, with other researchers, or along with the article, as well as adding the data to a repository for others to use. For each statement, participants indicated how problematic this was for them on a 7-point Likert scale, ranging from *totally disagree* (1) to *totally agree* (7). The scores were reversed so that a higher score meant more open to sharing the data. The parents were most favorable to sharing the data with colleague researchers (*M* = 4.34, *SE* = 0.16), but least open to placing the data in an open repository (*M* = 3.16, *SE* = 0.14). These results indicate that parents see some value in other scientists reusing the data but are reluctant to have the (anonymized) data of their child stored as open data to improve accountability, transparency, and reusability in science.

## Discussion

The results of this study indicate that parents perceive active consent procedures as appropriate in all types of research. How appropriate parents perceive passive consent procedures depends strongly on the vignette. That is, the ratings for passive consent varied between different types of research and research methods. In the total sample, we found that passive consent was reasonably as appropriate as active consent in *observation* studies and *co-creation* sessions. In addition, in the sample of parents with a child in secondary school, the appropriateness of passive consent was equivalent to active consent in *focus groups* and *(longitudinal) questionnaire* studies. This suggests that parents of children in secondary school are more lenient towards passive consent. Considering its advantages, this could warrant the use of passive consent procedures in these types of research studies in secondary school, depending on the situation regarding the institutional review board (IRB) and national legislation.

Moreover, the results of the exploratory analyses suggest that the appropriateness of passive consent coincides not only with the type of research, but also with the type of questions that are being asked within the study. The results align with the notion of Esbensen et al. (1996) that society has provided many safeguards for protecting this vulnerable target group and no additional precautions are necessary to protect children from questions about topics they frequently encounter in class, at home, in the media, or in their peer groups (Esbensen et al., 1996). As long as the questions do not deviate from these and do not ask about identifiable information, parents are open to passive consent procedures.

Irrespective of the consent procedure, parents indicated a strong liking for digital communication (i.e. email or website), preferably via the existing communication channel of the school. On the one hand, using this existing channel makes it more likely that parents will receive and read the information, which is especially important in passive consent procedures. On the other hand, it might also signal a form of familiarity and parents trust research that will be carried out within the school more.

Given these results, we advise research to collaborate with schools in creating digital communications to provide concise information in an attractive format. Over time, the length of the consent letter has increased dramatically, averaging a length of one page in 1970 to five pages in 2010 (Albala et al., 2010). This has a detrimental impact on content exposure and comprehension and makes it less likely that people will actually be informed. Moreover, it is plausible that this is one of the reasons of the biased samples as reviewed in the introduction. To break this trend, we recommend short digital communication with parents that help in obtaining *understood* consent (Isles, 2013). Notwithstanding the limited success of incorporating multimedia within the consent procedure assessed two decades ago (Flory & Emanuel, 2005), we believe that short videos can help to convey the information better and are more inclusive than their printed counterparts (Green et al., 2022). For example, different languages could be added as subtitles. Increasing the number of parents that are exposed to the information about the study could help with higher response rates in active consent procedures and more confidence that the parent has received and understood the information in passive consent procedures. Besides incentivizing schools, parents, and participants (Secor-Turner et al., 2010; Wolfenden et al., 2009), another promising avenue is to directly inform parents during parent-teacher conferences (Stein et al., 2007). So, especially for passive consent procedures, we advise using these meetings to inform the parents and following up with a short multimedia-enhanced information letter and an effortless opt-out procedure.

### Strengths, limitations, and future directions

Until now, the academic debate on parental consent procedures has mainly considered legislation and its effects on the composition of the sample. The strength of this study is that it adds the perspective of the parents to this debate, creating new insights about relevant stakeholders. On the one hand, the study reinforces active consent procedures, given that parents viewed active consent as appropriate for all types of research. On the other hand, including the parents’ perspective made a case for passive consent in some more specific cases. This could be used in discussions about regulation and legislation at an institutional, national, and international level. A second strength is that we used video vignettes and supportive visuals to study different types of research. We believe that the videos were easily accessible, inclusive, and engaging for participants. Therefore, the scenarios were clearer than merely textual explanation, and making the participation more pleasant for parents.

However, we also need to address some limitations. First, we introduced both procedures in an instruction video at the beginning of the study. Given the high appropriateness reported for active consent, we do not know what the participants were considering in their decision. Perhaps they did not consider the potential extra work involved in obtaining active consent. Second, in hindsight, we think that using the term *diary* study may have sounded too intimate for the participants, with the connotation differing for parents and researchers. This would explain the low appropriateness of passive consent for this method, compared to related research types such as *longitudinal questionnaires*. In similar vein, the wording of the vignettes might have had an effect on the specific ratings. For example, it is conceivable that parents were sensitive to wording that indicated a higher workload for the child or the degree to which the data collection is anonymous.Pre-testing the wording in the vignettes is advised for future studies. Third, the sample of parents in this study might be biased because they were participating in an academic study. Before starting the survey, the participants provided active consent and the university’s name and logo were visible. However, the sample was recruited by a panel agency and the bias might be less than when a university representative approaches participants directly. Finally, this study was conducted in only one country. It is plausible that parents from other nations hold a different perspective on providing consent for their children, due to different local legislation, culture, or views on the role of schools or the importance of research. Therefore, we invite fellow scholars to use the materials that we are sharing to replicate this study in other countries or cultures.

A remaining question that this study does not answer is how either active or passive consent procedures can affect the perceived sensitivity of the corresponding study. It is conceivable that parents attribute more importance (e.g. believing that more sensitive or more invasive data will be collected) to active consent procedures because they have to provide a signature agreeing to their child’s participation. In other words, when presented with a passive consent procedure, parents could assume that less sensitive data will be gathered because no active consent is required. Future studies should investigate this difference in perception to make a better judgment regarding when to opt for a specific consent procedure. Moreover, the high number of studies that use active consent procedures reinforces the assumption that children are not capable of comprehending and consenting to participate in academic research (Liu et al., 2017). However, there are indications that children as young as nine years old already possess a similar capacity to express their reasonable preferences regarding treatment in healthcare settings, and 12 to 14-year-olds have the same competencies as adults in this regard (Hein et al., 2015; Weithorn & Campbell, 1982). This suggests that academia might underestimate that children (and adolescents in particular) have the capacity and, perhaps even more importantly, the right to make an informed decision about participating in a study autonomously. Therefore, we would like to see more future work incorporating and investigating the self-determination of children in academic research (Isles, 2013; Livingstone, 2018; Waligora et al., 2014).

## Conclusion

Based on our results as well as associated literature, we conclude this paper by providing recommendations for when and how to use either active or passive parental consent for communication science research in the school context. We believe it is important to protect participants as much as possible, especially when they are children. But we do want to echo the notion of Liu et al. (2017) that we should not dogmatically apply active parental consent procedures for all studies that involve children. Instead, we should carefully balance the costs and benefits of both procedures for each specific study type and topic. Based on the input from the parents, we conclude our study with several suggestions. Our first is that whether informed parental consent is required in the first place should be determined. Depending on the physical location of the study and the data storage, the processing of non-personally identifiable information can be permissible for youths older than 13 (Livingstone, 2018). Or, when the study is part of the curriculum, *in loco parentis* consent might suffice. This means that a school representative (e.g. principal, dean, or headteacher) takes on the responsibility of the parents to provide consent for participation (Homan, 2001). Second, we advise using active parental consent as a default for studies in primary school. Exceptions to this would be studies involving only group-level processes where no personally identifiable information is recorded, such as observational studies or co-creation sessions. In secondary school, we advise using passive consent procedures for all research in group settings (i.e. observation, co-creation, and focus groups), and standardized (longitudinal) questionnaires. Our last suggestion is that in all cases, the researchers should make all reasonable efforts to inform the parents and children to obtain *understood* consent (Isles, 2013). We advise that parents should be informed in a face-to-face setting (e.g. parent-teacher conferences). Then, the procedure should be followed up by sending a short (i.e. one-page) consent letter, complemented by short videos and supplementary materials, via the school (digital) communication channels. Finally, the participants themselves should be involved and empowered in deciding if and how they want to participate in research in the school context (Waligora et al., 2014).
